# Multidrug-Resistant *Mycobacterium tuberculosis* Strain from Equatorial Guinea Detected in Spain

**DOI:** 10.3201/eid1511.090449

**Published:** 2009-11

**Authors:** Patricia Gavín, María J. Iglesias, María S. Jiménez, Laura Herrera-León, Elena Rodríguez-Valín, Nalin Rastogi, Josefa March, Rosa González-Palacios, Elia Palenque, Rafael Ayarza, Elena Hurra, Isolina Campos-Herrero, María A. Vitoria, María A. Lezcano, María J. Revillo, Carlos Martín, Sofía Samper

**Affiliations:** Instituto Aragonés de Ciencias de la Salud, Zaragoza, Spain (P. Gavín, S. Samper); Hospital Universitario Miguel Servet, Zaragoza (P. Gavín, S. Samper, M.A. Lezcano, M.J. Revillo); Centro de Investigación Biomédica en Red Enfermedades Respiratorias, Madrid, Spain (P. Gavín, S. Samper, M.J. Iglesias, C. Martín, M.A. Lezcano, M.A. Vitoria, M.J. Revillo); Universidad de Zaragoza, Zaragoza (M.J. Iglesias, C. Martín); Instituto de Salud Carlos III, Madrid (M.S. Jiménez, J. March, L. Herrera-León, E. Rodríguez-Valín); Centro de Investigación Biomédica en Red de Epidemiología y Salud Pública, Madrid (E. Rodríguez-Valín); Institut Pasteur, Guadeloupe, France (N. Rastogi); Hospital Universitario Príncipe de Asturias, Alcalá de Henares, Madrid (R. González-Palacios); Hospital 12 de Octubre, Madrid (E. Palenque); Hospital Galdakao-Usansolo, Galdacano, Vizcaya, Spain (R. Ayarza); Hospital de Cruces, Baracaldo, Vizcaya, (E. Hurra); Hospital Universitario de Gran Canaria Doctor Negrín, Las Palmas de Gran Canaria, Spain (I. Campos-Herrero); Hospital Clínico Universitario, Zaragoza (M.A. Vitoria)

**Keywords:** Tuberculosis, multidrug-resistant, Equatorial Guinea, transmission, letter

**To the Editor**: Eleven years of molecular epidemiologic data allowed the Spanish Multidrug-resistant Tuberculosis (MDR TB) Surveillance Network to identify a specific MDR *Mycobacterium tuberculosis* strain that had been imported into Spain from Equatorial Guinea (*1*). Our study brings to light the potential dissemination of this strain (named MDR-TBEG) in Equatorial Guinea, a country where little is known about the extent and features of TB or MDR TB. It also highlights that MDR strains can spread across continents, and thus MDR TB’s emergence in any country becomes a global problem.

Ten MDR *M. tuberculosis* isolates obtained from 10 patients from Equatorial Guinea were detected in Spain during 2000 through 2008. Evidence of clonality was found within the 10 isolates because all exhibited identical genetic profiles defined by different molecular epidemiology methods (*2*,*3*) and mutations involved in drug resistance (Figure). Notably, none of the remaining 504 MDR isolates in the Spanish database matched SIT177, a spoligotype belonging to the Latin American–Mediterranean 9 (LAM9) subfamily (*4*).

**Figure:**
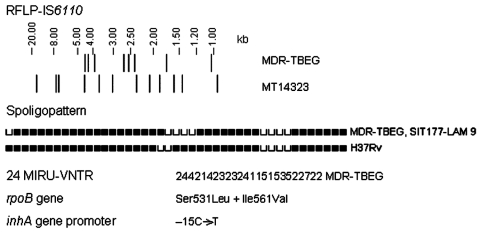
Genetic profile of the multidrug-resistant tuberculosis Equatorial Guinea (MDR-TBEG) strain. RFLP, restriction fragment length polymorphism; SIT, spoligotype international type; LAM, Latin American-Mediterranean; MIRU-VNTR, mycobacterial interspersed repetitive-unit variable-number tandem-repeat. MIRU-VNTR loci order: MIRU 02, VNTR 42, VNTR 43, MIRU 04, MIRU 40, MIRU 10, MIRU 16, 1955, MIRU 20, QUB-11b, ETRA, VNTR 46, VNTR 47, VNTR 48, MIRU 23, MIRU 24, MIRU 26, MIRU 27, VNTR 49, MIRU 31, VNTR 52, QUB-26, VNTR 53, MIRU 39.

The data routinely collected for all cases of MDR TB have been previously described (*1*). All 10 patients in the study were from Equatorial Guinea, a small African country on the Gulf of Guinea with a population of ≈500,000, an MDR TB rate >2.0% (*5*) of all combined (new and previously treated) TB cases, and an estimated adult HIV prevalence rate of 3.2% (www.who.int/globalatlas/predefinedReports/EFS2008/full/EFS2008_GQ.pdf). The MDR TB isolates were collected within a 9-year period (Technical Appendix): 1 in 2000, 2 in 2001, 3 in 2003, 1 in 2004, 2 in 2007, and 1 in 2008. According to their hospitals of origin, the patients were geographically dispersed in 6 different Spanish cities. We found that the interval between the patients’ arrival in Spain to the initiation of anti-TB treatment was <3 months in 6 patients, 3 of whom were clinically ill at the time of arrival. Seven patients were adult men, 2 were adult women, and 1 was an 8-year-old girl. The patients’ mean age was 30 years (range 8–54 years). Three patients were seropositive and 4 were seronegative for HIV infection (the HIV status of 3 patients was unknown). Data on prior anti-TB treatment was available for 7 case-patients, of whom only 1 had a history of antecedent TB chemotherapy. Altogether, 3 patients died before completing treatment, including 2 patients affected by miliary TB, 1 of whom was HIV-coinfected. The third patient who died was a student without a known history of immunosuppression or previous TB who had lived for 2 years in Spain. We could not establish any epidemiologic links between these patients during their stay in Spain.

Analysis of drug resistance genes showed that all isolates harbored the *inhA* promoter mutation –15C→T (*6*). Alterations in the *inhA* gene were previously reported in 80% of the isoniazid-resistant isolates from Equatorial Guinea (*5*). Notably, a double mutation in the *rpoB* gene affecting codons 531 (Ser531Leu) and 561 (Ile561Val) was detected in the 10 MDR isolates. The presence of this uncommon mutation, Ile561Val, outside the rifampin resistance–determining region supports the hypothesis that the MDR isolates are clonal in origin. Furthermore, we demonstrated the absence of Ile561Val mutation in 3 drug-susceptible *M. tuberculosis* strains with an SIT177-LAM 9 spoligotype pattern, which ruled out a relationship between this spoligotype and the Ile561Val mutation.

Further analysis with phylogenetic markers assigned MDR-TBEG to the principal genetic group 2, the Euro-American lineage of *M. tuberculosis* and its West African sublineage, on the basis of polymorphisms in codons *kat*G463 and *gyr*A95, the 7-bp pks15/1 deletion, and RD174 (*7*,*8*), respectively. The analysis of the RD^Rio^ deletion confirmed that the strain belongs to the major RD^Rio^ sublineage of the LAM *M. tuberculosis* spoligotype family (*9*). This sublineage is a major cause of TB in Rio de Janeiro (Brazil) but has disseminated globally. Additional information on the geographic distribution of SIT177-LAM 9 was obtained from the updated International Spoligotyping Database (SITVIT2) of the Institut Pasteur de Guadeloupe. SITVIT2 (consulted on 23 July 2008) contained 57 isolates belonging to SIT177. Almost 50% (n = 28) came from Brazil, and 14% from Africa (Morocco, n = 6; Senegal, n = 2). The remaining isolates with known countries of origin (n = 9) were distributed in other unrelated countries. These data indicate that this particular spoligotype pattern is widely distributed.

We identified 1 MDR strain of *M. tuberculosis* RD^Rio^ sublineage isolated in Spain from Equatorial Guinean patients. Although the transmission of MDR-TBEG in Spain could not be conclusively ruled out, the fact that MDR TB developed in most patients within 3 months after their arrival, as well as the spatiotemporal distribution of the MDR TB cases and its clonal origin, strongly suggest that MDR-TBEG was imported into Spain and that active transmission of this particular clone could be occurring in Equatorial Guinea. However, additional molecular and epidemiologic studies should be conducted in this sub-Saharan country to ascertain its role in recent transmission of MDR TB. Greater international efforts should be made to provide appropriate tools to resource-limited areas for fighting against MDR TB and preventing development of extensively drug-resistant TB.

## Supplementary Material

Technical Appendix Demographic and clinical characteristics of 10 patients from Equatorial Guinea infected with multiple drug resistant tuberculosis Equatorial Guinea strain*
