# Supplementary material for: Multidrug-Resistant Mycobacterium tuberculosis Strain from Equatorial Guinea Detected in Spain
Source: Emerg Infect Dis. 2009 Nov;15(11):1858–60. doi: 10.3201/eid1511.090449 (PMC2857231; doi:10.3201/eid1511.090449)
Supplement: Technical Appendix — Demographic and clinical characteristics of 10 patients from Equatorial Guinea infected with multiple drug resistant tuberculosis Equatorial Guinea strain* [file 09-0449_Techapp-s1.pdf]

Multidrug-Resistant *Mycobacterium tuberculosis* Strain from Equatorial Guinea Detected in Spain

Technical Appendix

Table. Demographic and clinical characteristics of 10 patients from Equatorial Guinea infected with multiple drug resistant tuberculosis Equatorial Guinea strain*

Patient no. †	Age, y/sex‡	Date of arrival in Spain	Date of treatment initiation	City of isolation	Resistance pattern§	TB type	Sputum smear¶	History of prior TB treatment	HIV serology
1	23/F	2000 Sep	2000 Nov	Madrid	HR	Pulmonary	NA	NA	NA
2	29/F	2000 May	2000 Jul 20	Madrid	HRES	Pulmonary	Positive	No	NA
3	8/F	EG	NA	Barakaldo	HRZ	Pott’s disease		NA	NA
4	54/M	2002 Nov	2003 Jan	Madrid	HRES	Miliary pattern	Positive	No	Negative
5	21/M	2001 Jun	2003 May	Las Palmas de Gran Canaria	HREta	Pulmonary	Positive	No	Negative
6	30/M	2002 Nov	2003 Oct	Alcalá de Henares	HRZEta	Miliary pattern	Positive	NA	Positive
7	27/M	1994	2004 Jun	Madrid	HR	Lymph node		No	Negative
8	49/M	EG	2008 Jan	Zaragoza	HR	Pulmonary	Positive	No	Positive
9	41/M	EG	2007 Sep	Alcalá de Henares	HREClaEtaRfb	Pulmonary	Negative	Yes	Positive
10	24/M	By the end of 2002	2008 Mar	Galdakao	HREta	Pulmonary	Positive	No	Negative

*NA, not available; EG, resident of Equatorial Guinea referred to a Spanish hospital for diagnosis and treatment.
†All patients were born in Equatorial Guinea.
‡Age in years.
§H, isoniazid; R, rifampin; E, ethambutol; S, streptomycin; Z, pyrazinamide; Eta, ethionamide; Cla, clarithromycin; Rfb, rifabutin.
¶Sputum smear not applicable in Pott’s disease or lymph node.
